# Txicological  Aspects of Newly Designed Macrocyclic Complexes of Iron(II)

**DOI:** 10.1155/MBD.2002.315

**Published:** 2002

**Authors:** Ashu Chaudhary, R. V. Singh

**Affiliations:** 1 Department of Chemistry University of Rajasthan Jaipur- 302004 India

## Abstract

Brine shrimp lethality of a new series of 16 to 26-membered macrocycles of iron(II) containing tetraaza groups and prepared by the template condensation reaction of diacarboxylic acids (malonic, succinic, glutaric or adipic) with 2,6-diaminopyridine and diethylenetriamine in 1:2:2 molar ratios have been studied. Structures and bonding of the macrocyclic complexes have been proposed based on elemental analyses, IR, electronic, X-ray and mass spectral studies. An octahedral geometry for these complexes has been proposed as the binding sites are the nitrogen atoms of the macrocycles. The formation of the complexes as [Fe(L^n^)Cl_2_] has been established on the basis of the chemical composition. The complexes have also been screened against several microbes.


### TOXICOLOGICAL ASPECTS OF NEWLY DESIGNED MACROCYCLIC COMPLEXES OF IRON(II)

Ashu Chaudhary and R. V. Singh*

Department of Chemistry, University of Rajasthan, Jaipur – 302004, India
< kudiwal@datainfosys.net > Fax : +91-141-519221

### ABSTRACT

Brine shrimp lethality of a new series of 16 to 26-membered macrocycles of iron(II) containing tetraaza groups and prepared by the template condensation reaction of diacarboxylic acids (malonic, succinic, glutaric or adipic) with 2,6-diaminopyridine and diethylenetriamine in 1:2:2 molar ratios have been studied. Structures and bonding of the macrocyclic complexes have been proposed based on elemental analyses, IR, electronic, X-ray and mass spectral studies. An octahedral geometry for these complexes has been proposed as the binding sites are the nitrogen atoms of the macrocycles. The formation of the complexes as $[Fe(L^n)Cl_2]$ has been established on the basis of the chemical composition. The complexes have also been screened against several microbes.

### INTRODUCTION

Studies on macrocyclic complexes have shown that some of them are involved in important biological processes, such as photosynthesis and dioxygen transport[1] in addition to their catalytic properties[2] which may lead to important industrial applications. Their enhanced kinetic and thermodynamic stabilities led to a widespread study of the features which also influence their potential applications as metal extractants[3] and as radiotherapeutic[4] and medical imaging agents[5]. Macrocyclic complexes are best prepared with the aid of metal ions as templates to direct the steric course of the condensation reaction which ultimately results in ring closure[6-8]. Different ring sizes of macrocycles are now readily available by convenient methods as already reported[9-16]. In most of the cases, high dilution techniques are employed for the cyclization process[12] when template is not operative. Macrocyclic polyamines have attracted increasing attention because of their unique property to form a very stable chelates with various heavy metal ions[17].

Many of new organic chemicals are prepared annually throughout the world and many of them entered into pharmacological screening to determine if they have useful biological activity[18] With this intention, we have selected four synthesized compounds of iron(II) having a tetraoxotetraaza ligand, formed by the template condensation of dicarboxylic acids (malonic, succinic, glutaric or adipic) with 2,6-diaminopyridine or diethylenetriamine in the presence of ferrous chloride.

Brine shrimp lethality bioassay is a recent development in the assay procedure[19] for the bioactive compounds, which indicates cytotoxicity as well as a wide range of pharmacological activity e.g., anticancer, antifungal, pesticidal, etc. Bioactive compounds are almost always toxic in high dose. Pharmacology is simply toxicology at a lower dose or toxicology is simply pharmacology at a higher dose. Thus in vivo lethality a simple zoological organism can be used as a convenient monitor for the screening of bioactive synthetic compounds.

### MATERIALS AND METHODS

The chemicals including malonic acid, succinic acid, glutaric acid, adipic acid, 2,6-diaminopyridine and diethylenetriamine were used as obtained from E. Merck. $FeCl_2.4H_2O$ was used without further purification.

**Synthesis of 6,8,14,16-dipyrido-2,4,10,12-tetraoxo-1,5,9,13-tetraazacyclohexadecane iron(II), chloride $[Fe(L^1)Cl_2]$**

$FeCl_2.4H_2O$ (1.01g/5.08mmol) was dissolved in methanol (25mL) and cooled in an ice bath. To this was added 2,6-diaminopyridine (1.10g/10.07 mmol) solution in methanol (25 mL) and put in magnetically stirred 100 mL round bottom flask. The reaction is followed by addition of malonic acid (1.05g/10.08mmol) in MeOH (25 mL). The resulting mixture was stirred for 24-25 hrs. The solid product was isolated by filtration, repeatedly washed with same solvent and dry in vacuo (yield, 49%). The compound was recrystallized in benzene and dried again in vacuo.

**Synthesis of 7,9,16,18-dipyrido-2,5,11,14-tetraoxo-1,6,10,15-tetraazacyclooctadecane iron(II) $[Fe(L^2)Cl_2]$**

$FeCl_2.4H_2O$ (1.12g/5.63mmol) in methanol (25 mL) and 2,6-diaminopyridine (1.23g/11.27 mmol) in methanol (25 mL) were added in a solution of succinic acid (1.33g/11.27 mmol) in methanol. This mixture was stirred continuously at room temperature for 24-25 hrs. The product obtained was washed with MeOH and dried in vacuo (yield, 37%). The compound was recrystallized in benzene.

**Synthesis of 8,10,18,20-dipyrido-2,6,12,16-tetraoxo-1,7,11,17-tetraazacyclodidodecane iron(II) chloride [Fe(L⁴)Cl₂]**

The procedure is same as described above. The reagents used were $FeCl_2.4H_2O$ (1.00g/5.03 mmol), 2,6-diaminopyridine (1.10g/10.07 mmol). The compound was recrystallized in dimethylformamide.

**Synthesis of 2,4,12,14-tetraoxo-1,5,11,15-tetraazacyclodoecane iron(II) chloride [Fe(L⁵)Cl₂]**

This compound was isolated by the procedure as detailed former. The reagents used were $FeCl_2.4H_2O$ (1.00g/5.03 mmol), diethylenetriamine (1.04g/10.08 mmol) and malonic acid (1.05g/10.08 mmol). The compound was recrystallized in benzene.

**Synthesis of 2,5,13,16-tetraoxo-1,6,12,17-tetrazacyclodidodecane iron(II) chloride [Fe(L⁶)Cl₂]**

This preparation was analogous to the above. Succinic acid was used in place of malonic acid. The compound was recrystallized in benzene.

**Synthesis of 2,6,14,18-tetraoxo-1,7,13,19-tetraazacyclododecane [Fe(L⁷)Cl₂]**

The preparation method was same as described former. The reagents used were $FeCl_2.4H_2O$ (1.07g/5.38 mmol), diethylenetriamine (1.11g/10.75 mmol) and glutaric acid (1.43g/10.82 mmol). The compound was recrystallized in benzene.

**Synthesis of 2,7,15,20-tetraoxo-1,8,14,21-tetraazacyclohexadoecane iron(II) chloride [Fe(L⁸)Cl₂]**

The compound was synthesized by the former method. The reagents used were $FeCl_2.4H_2O$ (1.01g/5.08 mmol), diethylenetriamine (1.05g/10.17 mmol) and adipic acid (1.55g/10.60 mmol). The complex was recrystallized in DMF.

The purity of the compounds was checked by T.L.C. on silica gel-G using anhydrous methanol and dimethylformamide (1:2) as a solvent. Each of the compound moves as a single spot indicating the presence of only one component and hence their purity.

**Analytical Methods and Physical Measurements**

Conductivity measurements were made with a systronic model 305 conductivity bridge in dry dimethylformamide. Molecular weights were determined by the Rast camphor method. IR of the soild samples were recorded as KBr discs on a Nicolet magna FT-IR 550 spectrophotometer. Electronic spectra in dimethylsulphoxide were recorded on a UV-160A, Shimadzer spectrophotometer in the range 200-600 nm using methanol as the solvent. X-Ray powder diffraction spectra of the compound was obtained on the philips model P.W. 1840 automatic diffractometer using Fe (Kα) target with Mg filter. The wavelength used was 1.9373 A° and the reflection from 5-65°C was recorded. The mass spectra of the compound was recorded on a JEOL FX 102/DA-6000 mass spectrometer/data system using Argon/Xenon (6 KV, 10 mA) as the FAB gas. The accelerating voltage was 10 KV and the spectra recorded at the room temperature. M-Nitrobenzyl alcohol was used as the matrix. Nitrogen and chlorine were estimated by Kjeldahl's and Volhard's method, respectively. Iron was estimated gravimetrically.

### RESULTS AND DISCUSSION

The physical properties and analytical data of the complexes are given in Table I. All the complexes are slightly soluble in common organic solvents but highly soluble in DMF and DMSO. The molecular conductance in anhydrous DMF are in the range 15-29 $ohm^{-1}$ $cm^2$ $mol^{-1}$ showing them to be non-electrolytes. Molecular weights of the complexes indicate the monomeric nature of the complexes. Elemental analysis agree well with the stoichiometry and chemical formula of the compound $[Fe(L^n)Cl_2]$.

**Table I : Physical Properties and Analytical Data of Fe (II) Macrocyclic Complexes**

| Compound | Emperical Formula | M.P. °C | Colour | Analysis (%) N Found (Calcd.) | Cl Found (Calcd.) | Fe Found (Calcd.) | Mol. Wt. (Calcd.) |
|---|---|---|---|---|---|---|---|
| $[Fe(L^1)Cl_2]$ | $C_{16}H_{14}O_4N_6Cl_2Fe$ | 192 | Dark Green | 16.59 (17.47) | 14.07 (14.74) | 10.99 (17.61) | 466 (481) |
| $[Fe(L^2)Cl_2]$ | $C_{18}H_{18}O_4N_6Cl_2Fe$ | 210 | Dark Green | 15.61 (16.51) | 13.39 (13.95) | 10.27 (10.97) | 486 (509) |
| $[Fe(L^3)Cl_2]$ | $C_{20}H_{22}O_4N_6Cl_2Fe$ | 141 | Dark Green | 14.66 (15.64) | 12.55 (13.20) | 9.84 (10.40) | 505 (537) |
| $[Fe(L^4)Cl_2]$ | $C_{22}H_{26}O_4N_6Cl_2Fe$ | 133 | Dark Green | 13.37 (14.87) | 11.94 (12.54) | 9.52 (9.98) | 529 (565) |
| $[Fe(L^5)Cl_2]$ | $C_{14}H_{26}O_4N_6Cl_2Fe$ | 139 | Bright Brown | 17.08 (17.92) | 14.51 (15.12) | 11.14 (11.91) | 451 (469) |
| $[Fe(L^6)Cl_2]$ | $C_{16}H_{30}O_4N_6Cl_2Fe$ | 168 | Bright Brown | 16.02 (16.91) | 13.68 (14.27) | 10.72 (11.24) | 474 (497) |
| $[Fe(L^7)Cl_2]$ | $C_{18}H_{34}O_4N_6Cl_2Fe$ | 157 | Bright Brown | 14.71 (16.01) | 12.91 (13.51) | 10.14 (10.64) | 503 (525) |
| $[Fe(L^8)Cl_2]$ | $C_{20}H_{38}O_4N_6Cl_2Fe$ | 126 | Bright Brown | 14.23 (15.20) | 12.10 (12.82) | 9.51 (10.10) | 532 (553) |

***SPECTRAL STUDIES***

**Infrared Spectra**

The infrared spectra of the starting materials and their metal complexes were studied and some important features may be summerized as follows :

The IR spectra of 2,6-diaminopyridine or dietylenetriamine and dicarboxylic acids show the bands due to hydroxyl and amino group, which disappear in corresponding metal complexes, indicating the condensation of amines with the dicarboxylic acids and formation of the proposed macrocyclic frame work. A broad band due to NH in case of diethylenetriamine appears almost at the same position ~3058 $cm^{-1}$ in metal complexes indicating that the group remains uncoordinated. The spectra of all the complexes show a medium intensity band at 3244-3271 $cm^{-1}$ which is assigned to $\nu$(NH) mode of the amide group[20] or the secondary amino group of the diethylenetriamine. The amide I, amide II, amide III and amide IV bands appear at 1672-1704, 1524-1570, 1212-1263 and 650-677 $cm^{-1}$, respectively[21]. Strong and sharp absorption bands appearing in the regions 2895-2923 and 1400-1442 $cm^{-1}$ in the complexes are assigned to the C–H stretching and C–H bending vibrational modes, respectively. The aromatic ring stretching appeared at 1648, 1527 and 1446 $cm^{-1}$. The presence of aromatic C–N bands in the complexes appeared in the region of 825-839 $cm^{-1}$. The spectra of the complexes derived from 2,6-diaminopyridine do not show any change in the pyridine ring which confirms that the nitrogen does not participate in the coordinaton[22]. The bands in the region 350-452 $cm^{-1}$ in the spectra of all the complexes may be attributed to the Fe-N stretching vibrations[23]. The Fe-Cl stretching vibrations have been assigned at 265-340 $cm^{-1}$ as reported by others also[24]. The infrared spectral data of the complexes are given in Table-II.

**Table II : IR Vibrational Frequencies ($cm^{-1}$) for Iron (II) Macrocyclic Complexes**

| Compound | $\nu$ (N–H) | Amide Bands | | | | (C–H) | | $\nu$ (C–N) | $\nu$ (Fe–N) | $\nu$ (Fe–Cl) |
|---|---|---|---|---|---|---|---|---|---|---|
| | | I | II | III | IV | Stretching | Bending | | | |
| $[Fe(L^1)Cl_2]$ | 3268 | 1684 | 1533 | 1246 | 677 | 2895 | 1442 | 830 | 452 | 324 |
| $[Fe(L^2)Cl_2]$ | 3250 | 1697 | 1524 | 1251 | 672 | 2911 | 1400 | 828 | 443 | 315 |
| $[Fe(L^3)Cl_2]$ | 3265 | 172 | 1548 | 1263 | 665 | 2899 | 1427 | 825 | 420 | 332 |
| $[Fe(L^4)Cl_2]$ | 3256 | 1704 | 1570 | 1260 | 650 | 2923 | 1433 | 839 | 429 | 326 |
| $[Fe(L^5)Cl_2]$ | 3244 | 1702 | 1565 | 1240 | 655 | - | - | - | 350 | 265 |
| $[Fe(L^6)Cl_2]$ | 3258 | 1687 | 1543 | 1272 | 660 | - | - | - | 356 | 294 |
| $[Fe(L^7)Cl_2]$ | 3267 | 1680 | 1555 | 1248 | 668 | - | - | - | 373 | 275 |
| $[Fe(L^8)Cl_2]$ | 3271 | 1696 | 1530 | 1234 | 658 | - | - | - | 363 | 300 |

**Electronic Spectra**

The electronic spectra of iron (II) tetraazamacrocyclic complexes exhibit a weak intensity band in the region 840-892 nm, which may be assigned to the $^5T_{2g} \rightarrow {^5E_g}$ transitions consistant with an octahedral geometry[25].

**$^{57}$Fe Mössbauer spectra**

The mössbauer spectra of the iron (II) complexes have been recorded. The chemical isomer shift values ($\delta$) relative to natural iron foil, which are sensitive to both the oxidation and spin states of iron, are in agreement with the structural assignments made on the basis of above spectral evidences. The value of isomer shift (0.25-0.30 mm $s^{-1}$) and quadrupole splittings (0.65 mm $s^{-1}$) at the room temperature are characteristic of six-coordinated low spin iron (II) complexes[26].

**Mass Spectra**

In the mass spectrum of the compound $C_{18}H_{34}O_4N_6Cl_2Fe$, the molecular ion peak appeared at m/z 564. The prominent fragments are at 532 for $[Fe(C_{16}H_{18}O_4N_6)Cl_2]^+$; 500 for$[Fe(C_{16}H_{18}N_6)Cl_2]^+$; 487 for $[Fe(C_{19}H_{23}O_4N_5)Cl_2]^+$; 485 for $[Fe(C_{17}H_{21}O_4N_3)Cl_2]^+$; and 406 for $[Fe(C_{12}H_{16}O_4)Cl_2]^+$; due to the loss of $C_4H_8$, $C_6H_8O_2$, $C_3H_3N$, $C_5H_5N_3$ and $C_{10}H_{10}N_6$, respectively. Another peak appeared at m/z 493 is due to the loss of two chlorine atoms from the parent ions.

**X-Ray Diffraction Spectra**

In order to ascertain the lattice dynamics of these compounds X-ray diffraction of the compound $[Fe(C_{20}H_{24}O_4N_6)Cl_2]$ has been recorded. The observed interplanar spacing values ('d' in A°) have been measured from the diffractogram of the compound and the miller indices h, k and l have been assigned to each d value and 2θ angles are reported in Table III. The results show that the compound, belongs to 'orthorhombic' crystal system having unit cell parameters as a = 32.872, b = 13.001 c = 19.850.

$\alpha = \beta = \gamma = 90°$, respectively, max. dev. of $2\theta = 0.9$.

**Table III : X-Ray Diffraction Data of the Compound $[Fe(C_{20}H_{24}O_4N_6)Cl_2]$**

| Peak No. | 2θ obs | 2θ calcd | d-spacing obs | h | k | l |
|---|---|---|---|---|---|---|
| 1. | 17.90 | 17.86 | 6.227 | 5 | 0 | 1 |
| 2. | 17.90 | 18.04 | 6.227 | 0 | 2 | 1 |
| 3. | 19.10 | 19.01 | 5.839 | 5 | 1 | 0 |
| 4. | 19.10 | 19.29 | 5.839 | 2 | 2 | 1 |
| 5. | 21.60 | 21.50 | 5.170 | 3 | 1 | 3 |
| 6. | 22.40 | 22.51 | 4.987 | 0 | 0 | 4 |
| 7. | 26.10 | 25.97 | 4.290 | 7 | 1 | 1 |
| 8. | 26.90 | 26.73 | 4.165 | 7 | 1 | 1 |
| 9. | 28.60 | 28.43 | 3.922 | 0 | 2 | 4 |
| 10. | 32.20 | 32.22 | 3.493 | 8 | 0 | 3 |
| 11. | 34.70 | 34.53 | 3.248 | 0 | 3 | 4 |
| 12. | 34.70 | 34.76 | 3.248 | 10 | 0 | 1 |
| 13. | 34.70 | 34.70 | 3.248 | 1 | 3 | 4 |
| 14. | 42.50 | 42.34 | 2.673 | 1 | 2 | 2 |

Refined Values, a = 32.872, b = 13.001 and c = 19.850, $\alpha = \beta = \gamma = 90°$ respectively
max dev. of 2θ = 0.9

The following structure can be suggested for Fe (II) complexes on the basis of spectral evidences and their monomeric nature:

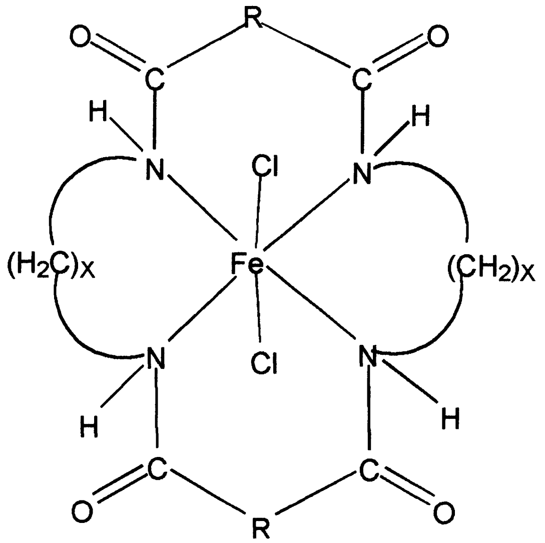

| | | | | | |
|---|---|---|---|---|---|
| $[Fe(L^1)Cl_2]$ | : | x=1 | R | = | $C_3H_5N$ |
| $[Fe(L^2)Cl_2]$ | : | x=2 | R | = | $C_3H_5N$ |
| $[Fe(L^3)Cl_2]$ | : | x=3 | R | = | $C_3H_5N$ |
| $[Fe(L^4)Cl_2]$ | : | x=4 | R | = | $C_3H_5N$ |
| $[Fe(L^5)Cl_2]$ | : | x=1 | R | = | $C_4H_9N$ |
| $[Fe(L^6)Cl_2]$ | : | x=2 | R | = | $C_4H_9N$ |
| $[Fe(L^7)Cl_2]$ | : | x=3 | R | = | $C_4H_9N$ |
| $[Fe(L^8)Cl_2]$ | : | x=4 | R | = | $C_4H_9N$ |

### MICROBIAL ASSAY

### Antifungal Activity

The antifungal activity of the compounds have been evaluated against *Macrophomina phaseolina, Fusarium oxysporum* and *Aspergillus niger* by the Radial Growth Method[27] using czapek's agar medium. The compounds were directly mixed with the medium in 50, 100 and 200 ppm concentrations. Controls were also run and three replicates were used in each case. The linear growth of the fungus was obtained by measuring the diameter of the fungal colony after four days. The amount of growth inhibition in all the replicates was calculated by the equation, Percent inhibition = C–T/Cx100, where, C is the fungal colony in the control plate and T is the diameter of the fungal colony in the test plate.

**Table IV : Fungicidal Screening Data of the Fe(II) Macrocyclic Complexes (Percent growth inhibition after 4 days at 25±2°C, Conc in ppm)**

| Compound | *Macrophomina phaseolina* | | | *Fusarium oxysporum* | | | *Aspergillus niger* | | |
|---|---|---|---|---|---|---|---|---|---|
| | 50 | 100 | 200 | 50 | 100 | 200 | 50 | 100 | 200 |
| $[Fe(L^1)Cl_2]$ | 69 | 87 | 89 | 81 | 89 | 92 | 78 | 86 | 88 |
| $[Fe(L^2)Cl_2]$ | 74 | 83 | 90 | 70 | 79 | 91 | 76 | 86 | 92 |
| $[Fe(L^3)Cl_2]$ | 72 | 80 | - | - | - | - | 76 | 82 | 90 |
| $[Fe(L^4)Cl_2]$ | 79 | 89 | 94 | 84 | 90 | 96 | 83 | 90 | 98 |
| $[Fe(L^5)Cl_2]$ | 51 | 72 | 88 | 50 | 71 | 92 | 47 | 74 | 90 |
| $[Fe(L^6)Cl_2]$ | 49 | 73 | 93 | 51 | 76 | 96 | 48 | 76 | 94 |
| $[Fe(L^7)Cl_2]$ | 52 | 68 | 82 | 49 | 60 | 84 | 47 | 68 | 90 |
| $[Fe(L^8)Cl_2]$ | 64 | - | - | 74 | 81 | 87 | 72 | 79 | 86 |
| Standard (Bavistin) | 82 | 100 | 100 | 85 | 100 | 100 | 86 | 100 | 100 |

**Antibacterial Activity**

The antibacterial activity of the compounds was evaluated by the Inhibition Zone Technique[28]. The compounds were tested against *Pseudomonas cepacicola, Staphylococcus aureus* and *Xanthomonas campestris*.

**Table V : Bacterial Screening Data of Fe (II) Macrocyclic Complexes (Percent growth inhibition after 24 hours at 28±2°C)**

| Compound | *Pseudomonas cepacicola* | | *Staphylocuccus aureus* | | *Xanthomonas campestris* | |
|---|---|---|---|---|---|---|
| | 500 | 1000 | 500 | 1000 | 500 | 1000 |
| $[Fe(L^1)Cl_2]$ | 4 | 13 | 6 | 8 | 8 | 13 |
| $[Fe(L^2)Cl_2]$ | 7 | 6 | 3 | 5 | 5 | 2 |
| $[Fe(L^3)Cl_2]$ | 5 | 8 | 4 | 7 | 6 | 9 |
| $[Fe(L^4)Cl_2]$ | 6 | 9 | 6 | 9 | 7 | 11 |
| $[Fe(L^5)Cl_2]$ | 7 | 10 | 9 | 11 | 9 | 12 |
| $[Fe(L^6)Cl_2]$ | 7 | 11 | 9 | 9 | 9 | 12 |
| $[Fe(L^7)Cl_2]$ | 5 | 9 | 4 | 6 | 5 | 2 |
| $[Fe(L^8)Cl_2]$ | 6 | 9 | 7 | 11 | 9 | 13 |
| Standard (Streptomycin) | 2 | 3 | 5 | 7 | 15 | 7 |

**Mode of Action**

The chelation theory[29] accounts for the increased activity of the metal complexes. The chelation reduces the polarity of the metal atom mainly because of partial sharing of its positive charge with the donor groups and posible π-electron delocalisation within the whole chelating ring. The chelation increases the lipophilic nature of the central atom which subsequently favours its permeation through the lipid layer of the cell membrane.

The degradative enzymes produced by the microorganism are important in host infection. For food deterioration and break down of organic matter. The enzyme production is here intended to mean both synthesis of the enzyme by the microorganisms and activity of the enzyme in the medium after it is produced. Since the metal complexes inhibit the growth of microorganism it is assumed that the production of enzyme is being affected and hence the microorganism is unable to utilize the food for itself or the intake of nutrients in suitable forms decreases and consequently the growth of microorganism is arrested, while higher concentration proves fatal. The higher concentration destroys the enzyme mechanism by blocking any of the metabolism path way and due to the lack of availability of proper food, the organism dies.

The results of biological activity have been compared with the conventional fungicide, Bavistin and the conventional bactericide streptomycin used as standards. The results achieved out of these studies have been enlisted in Tables IV and V in which the antifungal activity indicated that the metal chelates are more active than their parent amines and dicarboxylic acids. Similar trends were observed, in case of antibacterial activity.

**Brine Shrimp Lethality**

The eggs of Brine Shrimp, Artemia Salina (Leech) were hatched in a small tank divided by a net containing brine water. One part of the tank contained the eggs and on the other part, a light source was placed in order to attract the nauplii. Two days were allowed to hatch all the eggs and the nauplii were sufficiently matured for experiment[19].

The test compounds dissolved in DMSO were applied at five concentrations 5, 10, 20, 40 and 80 μ gm/ml. However not more than 50 μl of DMSO was added to nauplii in each vial. For each concentration, one vial containing the same value of DMSO plus brine water was used as a control group.

After 24 hours of incubation the vials were observed with the help of a magnifying glass and the number of survivors in each vial were counted and noted. From this data the mean percentage of mortality of the nauplii was calculated for each concentration.

**Table VI : Brine Shrimp Lethality of Macrocyclic Complexes of Fe(II)**

| Conc. Sample (C) μgm/ml | log C | Number of Applied Shrimps in I, II and III Vial | Number of Survivors in I, II and III Vial | % of Mortality in I, II and III Vial | Mean Value of Mortality | $LC_{50}$ μgm/ml |
|---|---|---|---|---|---|---|
| | | **Compound [$Fe(L^1)Cl_2$]** | | | | |
| 5 | 0.7 | 11,10,11 | 6,6,6 | 36.4,40.0,36.4 | 37.6 | |
| 10 | 1.0 | 10,10,10 | 6,6,5 | 40.0,40.0,40.0 | 43.3 | |
| 20 | 1.3 | 11,12,11 | 6,6,6 | 45.0,50.0,45.0 | 46.7 | 29.51 |
| 40 | 1.6 | 11,10,10 | 5,5,5 | 54.0,50.0,54.0 | 51.3 | |
| 80 | 1.9 | 10,10,11 | 4,4,5 | 60.0,60.0,54.5 | 58.2 | |
| Control | 0.0 | 10,11,10 | 10,11,10 | 00.0,00.0,00.0 | 00.0 | |
| | | **Compound [$Fe(L^2)Cl_2$]** | | | | |
| 5 | 0.7 | 10,10,10 | 6,6,7 | 45.5,45.5,50.0 | 36.7 | |
| 10 | 1.0 | 11,11,10 | 6,6,5 | 54.5,60.0,54.5 | 47.0 | |
| 20 | 1.3 | 11,12,11 | 5,6,5 | 60.0,60.0,63.6 | 56.3 | 14.15 |
| 40 | 1.6 | 10,10,11 | 4,4,4 | 40.0,40.0,30.0 | 61.3 | |
| 80 | 1.9 | 10,10,10 | 3,4,3 | 70.0,70.0,70.0 | 70.0 | |
| Control | 0.0 | 11,12,10 | 11,12,10 | 00.0,00.0,00.0 | 00.0 | |
| | | **Compound [$Fe(L^7)Cl_2$]** | | | | |
| 5 | 0.7 | 11,11,10 | 5,5,4 | 54.5,54.5,60.0 | 56.3 | |
| 10 | 1.0 | 10,11,10 | 4,4,4 | 60.0,63.6,60.0 | 61.2 | |
| 20 | 1.3 | 10,10,11 | 3,3,4 | 70.0,70.6,63.6 | 67.9 | 9.12 |
| 40 | 1.6 | 11,11,11 | 3,3,3 | 72.7,72.7,72.7 | 72.7 | |
| 80 | 1.9 | 11,11,11 | 2,2,2 | 81.8,81.8,81.8 | 81.8 | |
| Control | 0.0 | 13,10,11 | 13,10,11 | 00.0,00.0,00.0 | 00.0 | |
| | | **Compound [$Fe(L^8)Cl_2$]** | | | | |
| 5 | 0.7 | 10,10,11 | 5,5,6 | 50.0,50.0,45.6 | 48.5 | |
| 10 | 1.0 | 10,10,10 | 4,4,4 | 60.0,60.0,60.0 | 56.7 | |
| 20 | 1.3 | 11,11,10 | 3,3,3 | 63.6,63.6,60.0 | 62.4 | 5.75 |
| 40 | 1.6 | 10,10,10 | 2,2,3 | 70.0,70.0,70.0 | 70.0 | |
| 80 | 1.9 | 10,10,10 | 4,4,5 | 80.0,80.0,70.0 | 76.7 | |
| Control | 0.0 | 10,10,11 | 10,10,11 | 00.0, 00.0, 00.0 | 00.0 | |

### Conclusion

The Brine Shrimp Lethality of the compounds [$Fe(L^1)Cl_2$], [$Fe(L^2)Cl_2$], [$Fe(L^7)Cl_2$] and [$Fe(L^8)Cl_2$], were performed. The $LC_{50}$ values for all compounds were calculated and are given in Table VI.

The rate of mortality of the nauplii was increased with the increase in concentration of each sample. A plot of Log of sample's concentration versus percentage of mortality showed a linear correlation. From the graph, the $LC_{50}$ values of the samples were calculated and they were found 29.51, 14.51, 9.12 and 5.75 μ gm/ml, respectively.

Drug activity depends on the size, shape and degree of ionization of the drug molecule normally. It is found that the specific type of biological activity of a molecule is dependent upon more than just one functional group. Consequently, the addition of a single functional group to an inert organic substance doses not ordinarily imbue a molecule with a specific biological activity since more than one functional group normally is required for potent activity.

From this observation we conclude that compounds [$Fe(L^1)Cl_2$] and [$Fe(L^2)Cl_2$] have got little cytotoxic activity than that of compounds [$Fe(L^7)Cl_2$] and [$Fe(L^8)Cl_2$]. Among the compound [$Fe(L^1)Cl_2$] and [$Fe(L^2)Cl_2$], [$Fe(L^1)Cl_2$], showed less activity than [$Fe(L^2)Cl_2$]. Similarly a comparison of compounds [$Fe(L^7)Cl_2$] and [$Fe(L^8)Cl_2$] indicated that [$Fe(L^8)Cl_2$] is more active than compound [$Fe(L^7)Cl_2$]. From these observations we may conclude that combination of diethylenetriaminc make the compound [$Fe(L^8)Cl_2$] highly toxic for brine shrimp nauplii.

The compound [$Fe(L^2)Cl_2$] got significant antimicrobial activity but showed less cytotoxicity, therefore, the compound has potentiality to be a safe and effective antibiotic. But further extensive investigations on higher animal model is necessary to study it's other toxic effects.

**ACKNOWLEDGEMENT**

The authors are thankful to CSIR, New Delhi for financial assistance in the form of SRF vide Grant No. 9/149(288)/2K2EMRI.

**REFERENCES**

1. J.J.R. Frausto da silva and R.J.P. Williams, "The Biological Chemistry of the Elements," Clarendon Press, Oxford (1971).
2. N.R. Champness, C.S. Frampton, G. Geid and D.A Toucher, *J. Chem. Soc., Dalton Trans.*, 3031 (1994).
3. A.R. Adam, M. Antolovich, D.S. Baldwin, P.A. Duckworth, A.J. Leong, L.F. Lindoy, M. McPartlin and P.A. Tasker, *J. Chem. Soc. Dalton Trans.*, 1013 (1993).
4. J.P.L. Cox, K.J. Jankowski, R. Kataky, D. Parker, M.A. Eaton, N.R. Beeley, A.T. Millican, A. Harrison and C. Walker, *J. Chem. Soc. Chem. Commun*, 797 (1989).
5. R.B. Lauffer, *Chem., Rev.*, **87**, 901 (1987).
6. D.H. Bush, P.J. Jackson, M. Kojima, P. Chmielewski, N. Matsumoto, J.C. Stevens, W.Wu, D. Nosco, N. Berron, N. Ye, P.P. Warburton, M. Masarwa, N.A. Stephenson, G. Christopha and N.W. Alcock, *Inorg, Chem.*, **33**, 910 (1995).
7. D.C. McCollum, L. Hall, C. White, R. Ostrandes, A.L. Rheingold, J. Whelan and B. Bosnich, *Inorg. Chem.*, **33**, 924 (1994).
8. M.P. Suh, W. Skin, D. Kim and S. Kim, *Inorg, Chem.*, **23**, 618 (1984).
9. T.W. Bell and Papoulis, Angew, *Chem., Int. Edn. Engl.* **31**, 749 (1992).
10. G.H. Rothermel, L. Miao, A.L. Hill and S.C. Jackels, *Inorg. Chem.*, **31**, 4854 (1992).
11. M. Zinic and V. Skaric, *J. Org. Chem.*, **53**, 2582 (1988).
12. S.L. Rodgers, C. Yuen and K.N. Raymond *J. Am. Chem. Soc.*, **107**, 4094 (1985).
13. J.F. Carvalho, S. Hui Kim and C.A. Chang, *Inorg. Chem.*, **31**, 4065 (1992).
14. A.G. Schultz, D.J.P. Pinto and M. Welch, *J. Org. Chem.*, **53**, 1372 (1988).
15. H. Kataoka and T. Catagi, *Tetrahedron,* **43**, 4530 (1987).
16. R.W. Statz and R.C. Staufer, *Chem., Commun.*, 1682 (1970).
17. I. Tabushi, Y. Taniguchi and H. Kato, *Tetrahedron Letters,* **12**, 1049 (1977).
18. J.N. Delgoda and A. William, Remer's Wilson and Gisvold's, Text Book of Organic Medicinal and Pharmaceutical Chemistry, 9$^{th}$ Ed., *J. B. Lippincott Company,* 1 (1991).
19. B.N. Meyer, N.R. Ferrigni, J.E. Putnam, J.B. Jacobsen, D.E. Nicholas and J.L. Mcaulghlin, Brine Shrimp : A Convenient General Bioassay for Active Plant Constituents, Plant Media, **45**, 31 (1982).
20. M.B.H. Howlader, M.S. Islam and M.R. Karim, *Indian J. Chem.*, **39A**, 407 (2000).
21. D.L. Arora, K. Lal, S.P. Gupta and S.K. Sahni, *Polyhedron*, **5**, 1499 (1986).
22. W.U. Malik, R. Bembi and R.D. Singh, *Polyhedron*, **2**, 369 (1983).
23. P.S. Mane, S.G. Shirodkar, B.R. Aribad and T.K. Chondhekar, *Indian J. Chem.*, **40A**, 648 (2001).
24. A. Singh, S. Chandra and S. Beniwal, *J. Indian Chem. Soc.*, 75, **84** (1999).
25. A.T. Baker, P. Singh and V. Vigncvich, *Aust. J. Chem.*, **44**, 1041 (1991).
26. H.A. Goodwin, *Coord. Chem. Rev.*, **18**, 314 (1976).
27. D. Singh, R.B. Goyal and R.V. Singh, *Appl. Organoment. Chem.*, **5**, 45 (1991).
28. N. Fahmi and R.V. Singh, *Bol. Soc. Chil. Quim*, **41**, 65 (1996).
29. A. Kumari, R.V. Singh and J.P. Tandon, *Phosphorous, Sulphur and Silicon*, **66**, 195 (1992).

**Received: March 15, 2002 – Accepted: March 29, 2002 – Accepted in publishable format: April 16, 2002**

